# Comparative Multi-Stage TG-DSC Study of K^+^, Na^+^, Ca^2+^ and Mg^2+^-Exchanged Clinoptilolite Forms

**DOI:** 10.3390/molecules30244770

**Published:** 2025-12-13

**Authors:** Tsveta Stanimirova, Nadia Petrova, Georgi Kirov

**Affiliations:** 1 Faculty of Geology and Geography, Sofia University “St. Kliment Ohridski”, 15 Tzar Osvoboditel Blvd., 1540 Sofia, Bulgaria; kirov.g@gmail.com; 2 Institute of Mineralogy and Crystallography—Bulgarian Academy of Sciences, 107 Acad. G. Bonchev Str., 1113 Sofia, Bulgaria; nadia5@mail.bg; 3 PERIMED, Central District, Vasil Aprilov Blvd. 15A, 4002 Plovdiv, Bulgaria

**Keywords:** multi-stage TG-DSC, clinoptilolite forms, water molecules type, molar hydration enthalpy

## Abstract

A multi-stage TG-DSC approach consisting of five heating/holding and five cooling/holding stages within one experiment in the temperature range 20–320 °C was applied to investigate the dehydration/hydration processes in K^+^, Na^+^, Ca^2+^, and Mg^2+^ clinoptilolite forms. The influence of extra-framework cations on the parameters characterizing these processes (such as mass changes, dehydration and hydration heats calculated per gram zeolite, amounts of water molecules leaving and entering the structure, and enthalpy values calculated per mol water) was established. The values of molar enthalpy of dehydration for different cationic clinoptilolite forms increase in different ways with temperature increasing (within the framework of 50–120 kJ mol^−1^). The data on the molar enthalpy are in good agreement with the distributions of the two types of water molecules—weakly bound to cations and water molecules coordinating cations in the applied crystal chemical models of the cationic exchange samples. The data obtained for water molecules and their molar enthalpies of dehydration for the various cationic forms are useful in studying the sorption of water vapor and other sorbates, in choosing a desiccant and an object to dry at room conditions, etc. The first data on the hydration energy of sequentially added water molecules in a dynamic cooling mode in the temperature range 320–20 °C were obtained.

## 1. Introduction

Clinoptilolite (Cpt) is the most widespread zeolite mineral with well-studied deposits and industrial mining on all continents. It has numerous applications in industry, agriculture, and ecology. Its properties and potential applications are the subject of intensive and diverse research [1,2,3,4,5]. Clinoptilolite is isostructural with heulandite, as they form a continuous isomorphic series with a general formula (Na_2_, K_2_, Ca, Mg, Sr, Ba)_x/2_ [Al_x_ Si_36–x_ O_72_]·yH_2_O, (x = 6 ÷ 9, y = 20–24) and HEU-type framework [6]. Samples with Si/Al < 4 have been considered heulandites, and those with a ratio > 4 clinoptilolites [7]. Ca^2+^ predominates as a charge-compensating cation in heulandites, while clinoptilolites are polycationic, with alkali cations dominating. The topology of the aluminosilicate framework and the positions of Ca^2+^, Na^+^, K^+^, Mg^2+^, and H_2_O in HEU-type structures have been established [8,9,10]. The seminal work of Ames, 1960 [11], on the ion-exchange selectivity series of the two minerals has revealed the potential of clinoptilolite for environmental applications such as the removal of Cs^+^, NH_4_^+^, Sr^2+^, etc. from waste solutions and for the modification of various properties of the samples [4,12].

The thermal properties of HEU-type minerals are the basis for distinguishing the two end members of the series: clinoptilolite and heulandite. The differences in their DTA curves and XRD patterns of heated samples are the most commonly used identification features. Clinoptilolite has high thermal stability (up to over 700 °C), while heulandite structure transforms into meta-heulandite-B at 230 °C [13]. The high thermal stability of clinoptilolite has been explained with the high Si/Al ratio in the framework [13], and/or with the predominance of alkali over alkaline earth cations [14]. According to Koyama and Takeuchi, 1977 [9], potassium cations in the center of an eight-membered tetrahedral ring prevent deformation and collapse of the framework.

The main and immediate effect of heating is the release of H_2_O molecules into the surrounding space. Some degrees of dehydration are also observed when zeolites are purged with dry air or N_2_, as well as when they are evacuated. Upon cooling, molecules smaller than the zeolite channel openings are selectively sorbed in the freed volume of the dehydrated samples, depending on their polarity (molecular seeding). Water molecules have a decisive advantage because of their large dipole moment, which is the basis of the application for drying gases, liquids, and solids [1,2,5].

Structural studies by X-ray and neutron diffraction have contributed significantly to the understanding of the desorption-sorption processes [15]. Powder X-ray diffraction studies have shown changes in the lattice parameters and cell volume during dehydration [16]. Single-crystal X-ray diffraction in situ [17,18] and ex situ [19,20,21] refinement have been revealed to have a certain sequence of evolving water molecules with the progress of dehydration and the resulting displacements of the extra-framework cations, and, in some cases, T-O-T changes. Structural and volumetric changes observed on clinoptilolite dehydration have been related to the type of extra-framework cations [22].

Because desorption is an endothermic process and sorption is an exothermic process, the measurement of the change in the mass of the samples and the absorption and release of heat during their course is carried out with different instruments and methods depending on the task of the study. These are variants of thermo-gravimetric analysis (TGA), differential thermal analysis (DTA), differential scanning calorimetry (DSC), and many other calorimetric methods. Clinoptilolite samples from different localities and their exchanged forms have been the subject of numerous DTA(DSC)-TG studies to clarify the role of exchangeable cations on thermal dehydration and stability [23,24,25]. Combining DSC-TG analysis with other analytical techniques, such as powder X-ray diffraction, Fourier transform infrared spectroscopy, and principal component analysis, has provided valuable insights into the mechanism of clinoptilolite dehydration [26]. Different gravimetric [27] and volumetric methods [28,29,30] have been used as well for the evaluation of dehydration-hydration processes. There are a variety of calorimetric techniques and methodology using so far for obtaining accurate hydration enthalpy values of several clinoptilolite or heulandite samples—HF solution calorimetry [31], immersion calorimetry [32,33,34], water vapor sorption calorimetry [35], and drop-solution calorimetry [36,37].

The calorimetric study of the clinoptilolite hydration energy finds applications in various fields in solving both environmental and technological problems, for example, (i) a huge amount of studies concerning the evaluation of the thermodynamic properties of clinoptilolites and their implications for the suitability of the Yucca Mountain site as a high-level nuclear waste repository have been conducted [34,38,39]. These studies show that the importance of zeolites extends far beyond their cation selectivity towards ^137^Cs and ^89^Sr, to phenomena affecting the entire thermo-hydrological system and predicted thanks to calorimetric studies. (ii) Calorimetric hydration heat measurements have been used to evaluate the natural clinoptilolite and their K, Na, Ca, and Mg ion-exchanged forms as exchangers for heat pump systems [33,35]. The efficiency of zeolite–water-adsorbed refrigeration/heat-pump systems has been compared with conventional heat pumps. Adsorption heat pumps are a serious alternative to mechanical heat pumps since they do not contain any hazardous materials for the environment. The main advantage of adsorption heat pumps is operation with thermal heat sources, particularly solar energy and waste heat [40,41,42].

The calorimetric techniques discussed so far can obtain hydration enthalpy values at a given temperature in one experiment. Quantitative enthalpies of dehydration could also be obtained through the TG-DSC approach in scanning mode [43]. An alternative approach using TG-DSC capabilities has been proposed for isothermal measuring of the heat of hydration in zeolites at a constant temperature of interest [44]. In our recent work, we aimed to test the capabilities of the TG-DSC method to obtain reliable data (values for heats of dehydration/hydration) by creating heating and cooling modes with holding moments within a single experiment [45,46]. A complex TG-DSC temperature profile (heating to 320 °C, holding, cooling to 40 °C, and then heating to 600 °C) has been arranged for studying Ca-clinoptilolite in static humid air and carrying dry air [45]. In this case, data for mass changes, heats of dehydration, and sorption of both water and nitrogen have been obtained. In later work [46], by complicating the experiment through several multi-stage experiments, consisting of a sequence of dynamic and isothermal modes during heating and the same during cooling in the specified temperature range, quantitative molar enthalpy data of dehydration (or re-hydration) for Ca-exchanged clinoptilolite have been obtained.

In the present work, we investigate the kinetics and energetics of water–zeolite interactions in different cationic forms (K, Na, Ca, and Mg-exchanged clinoptilolite) by applying the proposed multi-stage TG-DSC approach. We aim to clarify the role of individual exchange cations in the energetics of the dehydration/rehydration process, as well as the nature and quantitative distribution of water types in the zeolite forms. Based on TG-DSC and crystal chemical data, an attempt to evaluate the potential of clinoptilolite and its cationic forms as a desiccant and in other thermal engineering applications (heat storage, cooling, heat pumps, etc.) is made.

## 2. Results and Discussion

### 2.1. Standard TG-DSC Study

**Figure 1:**
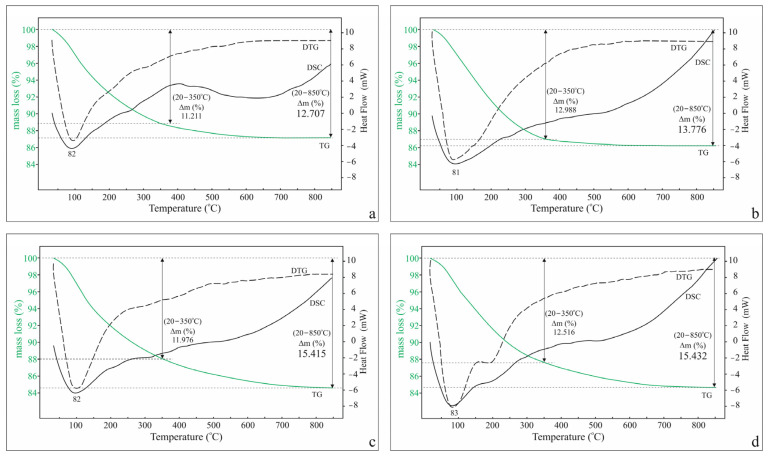
TG (DTG)-DSC curves in the temperature region 20–850 °C, Δm (%) is the mass loss of samples investigated in the mentioned temperature range: (**a**) K-Cpt; (**b**) Na-Cpt; (**c**) Ca-Cpt; (**d**) Mg-Cpt.

The mass loss data from TG analysis in the temperature range 20–850 °C reflect the specifics of the dehydration of individual cationic forms depending on their characteristic water contents. Cationic forms with monovalent extra-framework cations (K^+^ and Na^+^) are characterized by a significantly earlier completion of dehydration (at about 640 °C) in contrast to those with Ca^2+^ and Mg^2+^, in which mass loss ends at about 840 °C (Figure 1).

The nature of the TG curve shows a continuous course of the dehydration process with a maximum dehydration rate at about 80 °C. The profile of the DSC curve outlines two regions (25–400 °C and 400–850 °C) for the monovalent and three (25–150 °C, 150–400 °C, and 400–850 °C) for the divalent cationic forms.

### 2.2. Multi-Stage TG-DSC Study

The influence of the extra-framework cations on the parameters of dehydration and rehydration (sample mass changes, amounts of water molecules and their energies of release and addition, and others) was studied by applying a multi-stage experiment approach with alternating heating/holding and cooling/holding mode in the temperature range 20–340(320)–20 °C [46]. Because we study both processes (dehydration and rehydration) in one experiment, the segmented DSC peaks are in the endo- and exothermic direction, visualizing the dehydration and rehydration processes of the zeolite. The data provided by the multi-stage TG-DSC experiment allows us to determine several important parameters characterizing dehydration/rehydration processes of zeolites: the mass change (Δm, %) allows us to calculate the number of water molecules leaving or entering the structure during each stage. The values of heat released or received at each stage per gram of zeolite (Q_Z_, J g^−1^), calculated for the mass change per mol H_2_O for each stage, give the values for the enthalpy of dehydration or rehydration per mol H_2_O (ΔH, kJ mol^−1^). Due to the complexity of the experiments and their long duration, each of these experiments was only repeated. The experimental error for mass change values was found to be less than 1% for each stage, while that of the measured values for dehydration/rehydration heats depend on the dehydration/rehydration temperature, namely for the low temperature stages (in the range 20–200 °C) the error was found to be 3.0% for each stage and for the high temperature stages (in the range 200–320 °C)—about 4.5%.

#### 2.2.1. Four-Stage TG-DSC Study

**Figure 2:**
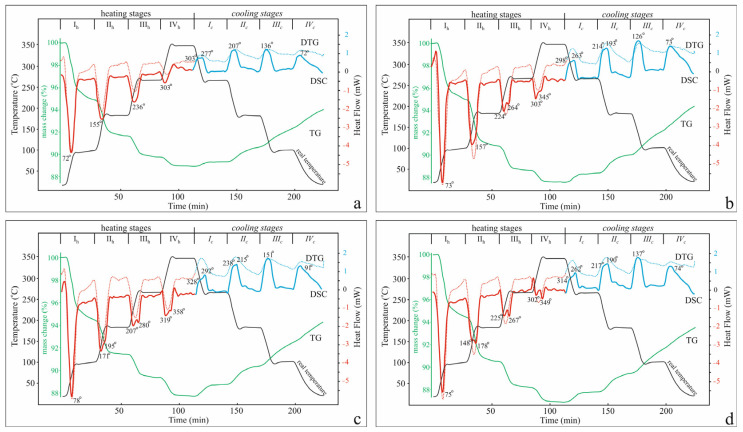
DSC-TG(DTG)-temperature time profiles from the experiment with both four heating and four cooling stages: (**a**) K-Cpt; (**b**) Na-Cpt; (**c**) Ca-Cpt; (**d**) Mg-Cpt.

This study was conducted in the temperature range 20–340 °C by alternating four stages of heating/holding and cooling/holding through 80 °C, with the holding time being 20 min (Figure 2). The temperature range was chosen as economically justified and covering the main area of dehydration. Within the framework of this chosen multi-stage experimental arrangement, the first four stages of dynamic heating/isothermal mode are associated with the dehydration of the zeolite, while the remaining four stages in dynamic cooling/isothermal mode are associated with the rehydration of the activated sample.

The mass loss data in this experiment (Table 1) show that for the monovalent cationic forms, dehydration at a temperature of 340 °C is almost complete (92% and 96% for K-Cpt and Na-Cpt, respectively), while for the divalent forms, it is 81% for Ca-Cpt and 85% for Mg-Cpt. The data obtained is in good agreement with the mass losses measured from the curves of conventional TG-DSC analysis in the temperature range 20–850 °C (Figure 1). The mass increase in the cooling mode is a consequence of the return of water molecules to the structure (hydration) from the air environment in the apparatus. Under the conditions of the experiment, the degree of rehydration for the monovalent cationic forms of Cpt relative to the dehydrated samples is 43.5% and 51% for K-Cpt and Na-Cpt, respectively. In the forms with divalent extra-framework cations, the degree of rehydration is slightly higher, for Ca-Cpt it is 53.5%, and for Mg-Cpt it is 53% (Table 1) in accordance with the higher values of the ionic potential of both cations.

The diffuse (weakly structured) shape of the DSC curves from standard heating (Figure 1) suggests a smooth, continuous nature of the dehydration process. The registered DTG curve in the four-stage experiment corresponds to a continuous process of dehydration (as well as hydration), while the course of the associated heat flow shows inhomogeneity in certain temperature ranges. On the DSC curves in some of the temperature regions (heating and cooling stage), a sharp splitting of the endothermic and exothermic peaks is recorded, which suggests that within the range, the processes are associated with the leaving (or entering) of water molecules that differ in energy. Temperature ranges in which splitting is observed, as well as the splitting depth, are influenced by the type of extra-framework cations. The water molecules interact in different ways with different extra-framework cations, which have been established by crystal structural study of clinoptilolite samples [19,20]. Thus, it could be assumed that the observed splitting reflects the presence of differently bound water molecules and marks the temperatures at which the release of these water molecules has different enthalpy values. However, the splitting of the endothermic (or exothermic) peaks does not allow accurate determination of the molar enthalpy of the processes. To solve these two problems, several experiments with different numbers of stages were carried out. It was found that for the cationic forms of the studied clinoptilolite, symmetrical and homogeneous DSC effects for each step are seen in a five-stage heating/holding and five-stage cooling/holding experiment, which allows determining the desired parameters.

#### 2.2.2. Five-Stage TG-DSC Study

**Figure 3:**
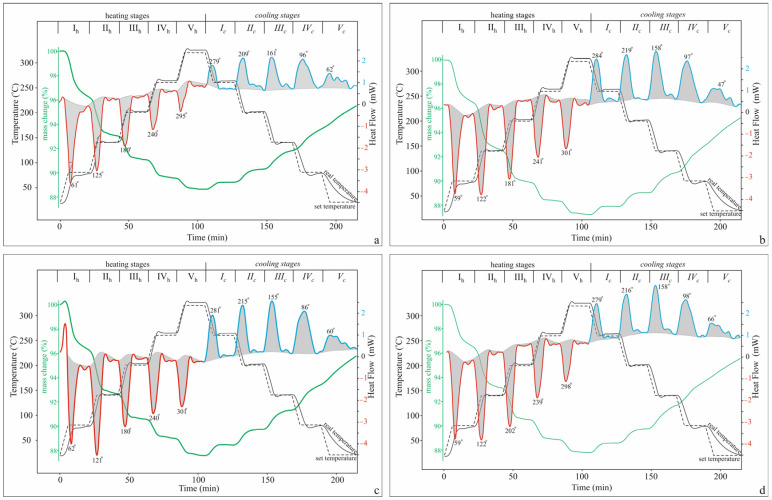
TG-DSC-temperature time profiles from the experiment with both five heating (red curve) and five cooling (blue curve) stages: (**a**) K-Cpt; (**b**) Na-Cpt; (**c**) Ca-Cpt; (**d**) Mg-Cpt.

Based on the data obtained from a multi-stage experiment with five heating/holding stages and five repeated cooling/holding stages (Figure 3), the dehydration and rehydration processes for the individual cationic clinoptilolite forms were separately examined in detail.

- Dehydration

The data obtained for the total mass loss (Δm, %) in the temperature range of the five-stage experiment (Table 2) are comparable to those obtained from the standard TG-DSC analysis (20–850 °C) for the studied temperature range (20–320 °C) (Figure 1). The obtained slightly higher values for the mass loss are due to the longer duration of the multi-stage experiment due to the inclusion of the holding mode. For all forms, the share of the isothermal mode for the mass loss is about 10–11% of the mass loss of the entire studied range (Table 2), approximately the same as the difference between the standard and 5-stage experiments. Unlike the standard DSC analysis, where the highly diffuse shape of the endothermic peaks makes it impossible to measure the heats of evolving volatiles, the multi-stage experiment allows for measuring the heats of dehydration per gram of zeolite (Qz) for each stage and mode (Table 2).

**Figure 4:**
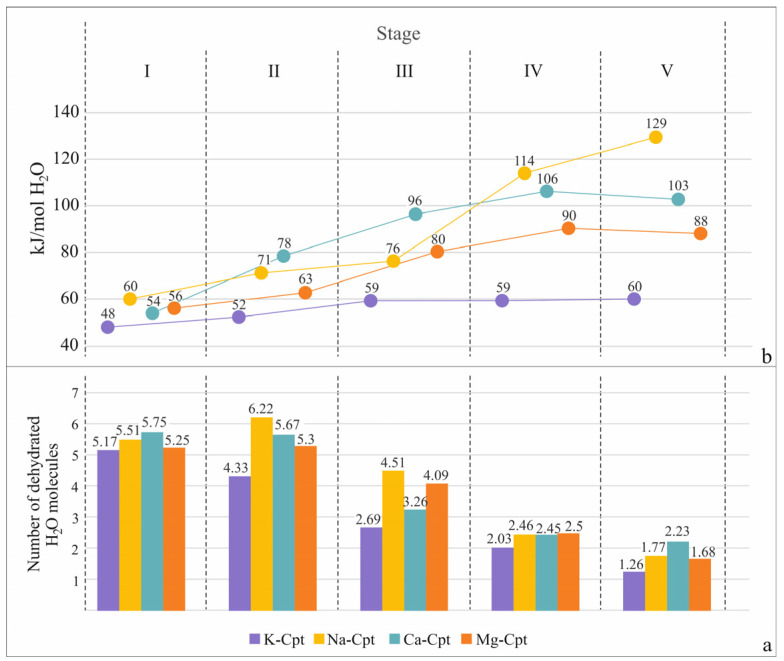
Number of dehydrated H_2_O molecules (**a**) and molar enthalpy of dehydration (**b**) for cationic exchanged Cpt forms.

For a more detailed characterization of the dehydration, the released water molecules and the molar enthalpy of dehydration are calculated for each temperature interval (Figure 4). The degree of dehydration for different samples varies widely: for samples with predominantly monovalent extra-framework cations, it is 90% (K-Cpt) and 94% (Na-Cpt), while for samples with divalent cations, it is lower (Ca-Cpt—81% and Mg-Cpt—79%). A comparison of the dehydration process occurring within the different stages shows the release of more water molecules in the first stages of dehydration compared to the final stages, regardless of the extra-framework cations (Figure 4a). At the same time, in terms of energy, an increase in the values of the molar enthalpy of dehydration from the I_h_ to V_h_ stage is established for all cationic forms, but the specific values and the range of change in the individual stages are strongly influenced by the type of cation (Figure 4b).

The dehydration molar enthalpy of samples with divalent compensating extra-framework cations (Ca-Cpt and Mg-Cpt) has a similar character of change in values with increasing temperature, with the values for Ca-Cpt being relatively higher than those for Mg. In contrast, for samples with monovalent extra-framework cations (K-Cpt and Na-Cpt), very different values for the molar enthalpy of dehydration are observed. In the K-Cpt form, the release of almost all water molecules in the entire temperature range studied showed that the same molar enthalpy is registered, which suggests a similar interaction of water molecules with both the compensating K^+^ and the framework. In the Na-Cpt sample, the release of 80% of water molecules is associated with enthalpy values from 60 to 76 kJ mol^−1^, while the release of the remaining 20% of water molecules is characterized by twice as high enthalpy values.

**Figure 5:**
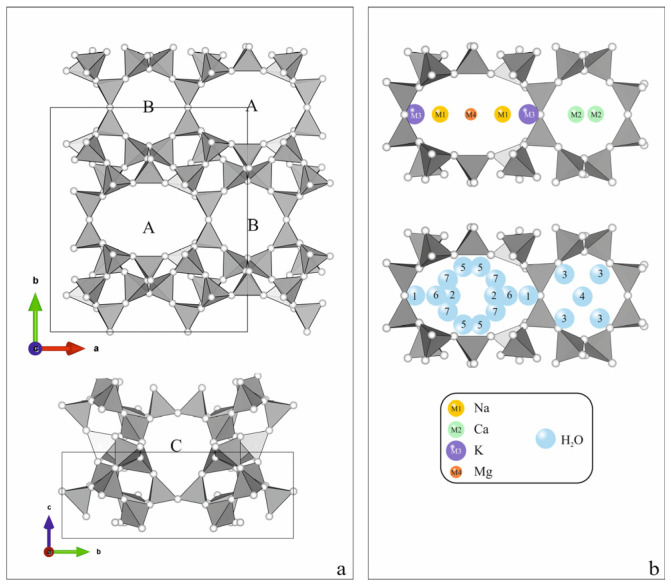
HEU-type tetrahedral framework projected along (**a**) [001], [100], [010]; (**b**) location of the extra-framework positions in the tetrahedral rings A and B.

**Figure 6:**
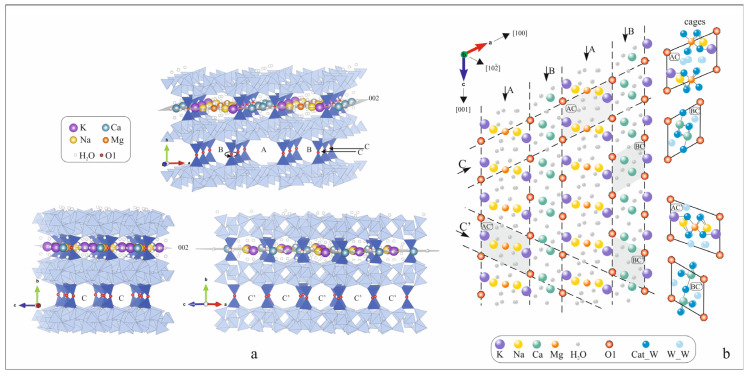
Gallery representation of the HEU type structure projected along (**a**) [001], [010], [10−2]; (**b**) distribution of O1 atoms in the plane 020, with directions of A, B, C, and C’ and location of the extra-framework cation positions in cages.

To explain the observed different thermal behavior of the individual forms, we take into account the crystal chemical features of the clinoptilolite structure, which is a HEU-type. The structural topology of the tetrahedral HEU framework is usually described as possessing space group C2/m (no. 12) with unit cell dimensions a = 17.6 Å, b = 17.9 Å, c = 7.4 Å, and β = 116.50°, and channels confined by ten-member and eight-member tetrahedral rings parallel to the c-axis (channels A and B). The eight-member ring channel running parallel to [100] (channel C) cross-links the former channels in a two-dimensional channel system parallel to (010), resulting in a layer-like structure. Chanel C is built from the eight-member ring C and is parallel to [100]. The projection of the tetrahedral rings on (001) and (100) is shown in Figure 5a with their notation according to Koyama and Takeuchi [9]. The apertures of channels A, B, and C are about 7.6 × 3.0 Å, 4.6 × 3.3 Å, and 2.9 × 4.7 Å, respectively [6]. All of them are bigger than the diameters of the water molecules and most of the mono- and bivalent cations, and in this way are occupied by the extra-framework cations and water molecules (Figure 5b). Because all the extra-framework cations are in one plane (020), we have proposed [47] a representation of the porous space in the aluminosilicate framework as a unified, continuous gallery structure between two tetrahedral layers “leaning” on parallel rows of “diortho” groups of T2 tetrahedra, connected by a common oxygen atom O1 (Figure 6a). The gallery space can be decomposed into four systems of parallel cages, notated on Figure 6b as AC, BC, AC’, and BC’, following the notation of the tetrahedral rings that enclose them and the lanes that cross into them. Two of these cages, BC and AC, have been marked as Cage I and Cage II [48]. Based on the analysis of published powder diffraction data, the possible local extra-framework cation–water assemblages (denoted as Cat_W in the present paper) and water assemblages (denoted as W_W in the present paper) and their interactions were determined. The water arrangement is based on the inter-atomic distances, occupation, and the opportunities to simultaneously occupy adjacent positions determined by the structural refinement, as well as the properties of cations (a charge, dimensions, hydration characteristics) and other considerations [47]. For example, the K position, regardless of whether it is in cage AC or AC’, always has only one water molecule coordinated with K^+^ (Figure 6b). Na^+^ cations, in addition to their own positions (Figure 6), also occupy those of Ca^2+^.

Depending on the distribution of Al^3+^ in the tetrahedra, Na^+^ cations in their own positions (cage AC’) can coordinate a different number of water molecules (2, 4, or 5), and in the Ca-position (cages BC and BC’), they are coordinated with 3 H_2_O (Figure 6b). Due to their small size, high charge, and, respectively, higher ionic potential, divalent cations coordinate more water molecules. Ca^2+^ is coordinated by 5 H_2_O in both positions it occupies, while Mg^2+^ is always coordinated with 6 H_2_O molecules (Figure 6b).

Based on this model, the number of water molecules, closely coordinating with different cations, is as follows: each K^+^ is coordinated with one H_2_O; Na^+^—average with three H_2_O; Ca^2+^—five H_2_O, and Mg^2+^—six H_2_O (Figure 6b).

Using the representations and quantitative distributions of the two types of water molecules—weakly bound to the cations (W_W) and water molecules coordinating the cations (Cat_W)—the total theoretical amounts (W_W and Cat_W) of water molecules and their theoretical quantitative distribution for the specific cation forms studied in the present study are calculated (Table 3).

The obtained mass losses (expressed as the number of water molecules) in the different stages are consistent with the concepts of the distribution of water molecules according to the above-described structural model (Table 4). In this agreement, we assume that the water molecules coordinated around each cation (denoted as Cat_W) leave sequentially, one by one. In addition, the notation “Cat” unites all non-skeletal cations presented in the structure, and the indices 1, 2, 3, 4, 5, and 6 after Cat_W denote the sequential number of the leaving (or remaining) water molecule.

The analysis of thermal data in the light of crystal chemical concepts about the distribution of water molecules in the structure, depending on the extra-framework cations, provides a good explanation for the observed differences in both the quantitative ratios of the leaving water molecules and mostly the values of the molar enthalpies. In the K-Cpt form, the large size, low charge, and ionic potential of K^+^ predetermine an almost equal number of coordinating (Cat_W) and less strongly bound water molecules (W_W) and a similar, but not identical, value of the molar enthalpy. It is evident that when the water molecules coordinate the cation, leaving the structure, the molar enthalpy increases sharply (Table 4). In the case of Na-Cpt, a stronger interaction of the cation with the weakly bound water molecules is registered compared to the other cationic forms. This is most likely due to both the high ionic potential and the large number of Na^+^ cations, as well as their uniform distribution in the pore space. In the experiment with Na-Cpt, it is even more clearly seen that each increase in molar enthalpy is associated with the sequential release of one of the coordinating Na^+^ water molecules. The release of one of the coordinated water molecules clearly strengthens the interaction of the Na^+^ cation with the remaining two H_2_O molecules, which is marked by the slightly higher energy of evolving the second one. Complete dehydration of the pore space causes straining of the structure, redistribution of cations, and, at high temperatures, destruction of the structure [16,18]. For this reason, in the studied Na-Cpt sample, the release of the last water molecule coordinated by Na^+^ is marked by a sharp jump in the value of the molar enthalpy (Table 4).

In the samples with predominantly divalent extra-framework cations (Ca-Cpt and Mg-Cpt), in the selected temperature range (25–320 °C) about 65% (68% for Ca-Cpt and 62% for Mg-Cpt) of the water molecules coordinated to the cations leave the structure, which corresponds to complete dehydration of K and Na cations and the release of three water molecules coordinated by Ca and Mg cations (Table 4). The polycationic composition of both samples reflects the absolute values of the calculated molar enthalpies. For example, the smaller values for Mg-Cpt compared to those of Ca-Cpt are due to the different amounts of K and Na cations—a larger amount of K^+^ (characterized by low molar enthalpy values) and less of Na^+^. Although this makes it difficult to determine the exact molar enthalpies of H_2_O release coordinated with divalent cations, clear trends are observed, controlled by the type of cation. As with monovalent cations, the measured enthalpy values for the release of water molecules in the samples with divalent cations show an increase, reflecting the sequential release of water molecules coordinating the cations. In contrast to the samples with monovalent cations, for Ca-Cpt and Mg-Cpt, the molar enthalpy values are observed to be maintained over a larger temperature range, with even a slight decrease in values at the end of the experiment (Table 4), which is most likely due to the rearrangement of the remaining significant amount of water molecules in the in the relatively emptier free space.

Based on the data obtained from the experimental part with heating and subsequent holding stages through 60 °C, it can be summarized that dehydration occurs with the sequential evolving of water molecules with different interaction strengths with the framework and/or extra-framework cations. The amount of evolved molecules and their dehydration energy depend on the extra-framework cation specificities. In the forms with monovalent cations (K-Cpt and Na-Cpt), the influence of the ionic potential of the cation is clear. The large size of K^+^ and the correspondingly very small ionic potential (Z/R = 0.75) determine both the presence of a small amount of water molecules coordinated with the cation and their relatively weak interaction with the cations, which is almost comparable to that of weakly bound water molecules (Table 4). Unlike K^+^, Na^+^ cations are characterized by a higher ionic potential (Z/R = 1.02), which reverses the quantitative relationship of weakly bound water molecules to cation-coordinated ones from 60:40 (9 to 6.5 H_2_O molecules) in K-Cpt to 30:70 (5 to 15.5 H_2_O) in Na-Cpt (Table 4). The high ionic potential of Na^+^ cations is also manifested in the 10 kJ mol^−1^ higher energy for weakly bound water molecules and by 10-15 kJ mol^−1^ higher energy of evolving 2/3 of the coordinated water molecules, as well as in the two-fold higher energy of evolving the last one coordinated with the cation (Table 4).

The divalent cations Ca^2+^ and Mg^2+^ are characterized by a very high ionic potential (Z/R = 1.92 for Ca^2+^ and Z/R = 2.70 for Mg^2+^), which causes coordination of 5 and 6 H_2_O molecules with Ca^2+^ and Mg^2+^, respectively. On the other hand, however, the cations are two times less in number; therefore, in the Ca-Cpt and Mg-Cpt samples, the amount of weakly bound water molecules is significantly high—40–45% (Table 3). The influence of the high ionic potential can also be traced here, regardless of the more diverse composition and partial dehydration. Although under experimental conditions, half of the water molecules coordinated with the cations are released, and the measured values for the molar enthalpy of their evolution are higher (85–105 kJ mol^−1^) compared to those of the forms with monovalent cations (60–75 kJ mol^−1^) (Table 4). Similar values for the molar enthalpy of dehydration obtained by conventional DSC analysis (heating mod 5 °C min^−1^ in temperature interval 20–800 °C) have been reported [43] in the study of natural heulandite, Na_1.46_K_0.23_Ca_3.44_Al_8.55_Si_27.44_O_72·24.0_H_2_O. An increase in molar enthalpy from 50 to 87 kJ mol^−1^ has been found with a decrease in the remaining amount of water molecules in the structure of the sample [43].

**Figure 7:**
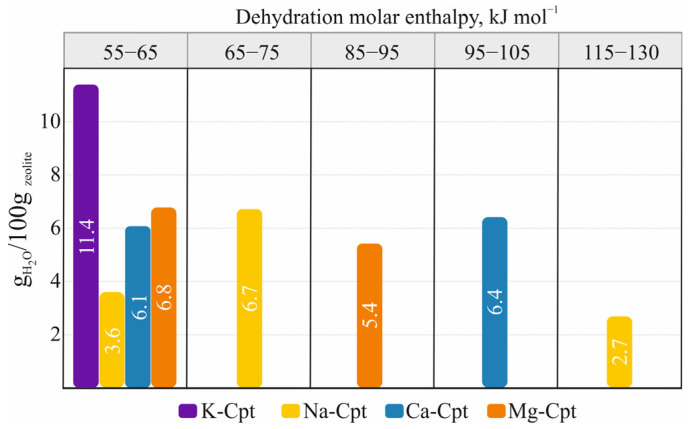
Quantitative distribution of water molecules with different dehydration enthalpies of the studied cationic forms.

The data obtained on the dehydration of different cationic forms in the temperature range 20–320 °C reveal possibilities for their practical application. For example, from the quantitative distribution of water molecules with different dehydration enthalpies for individual cationic forms (Figure 7) one can predict the value of the thermal energy stored in the dehydrated state, one can evaluate their qualities as water desiccants, which makes it possible to select objects for draying both by the amount of sorbate and by the strength of binding of water molecules, etc.

- Rehydration

**Figure 8:**
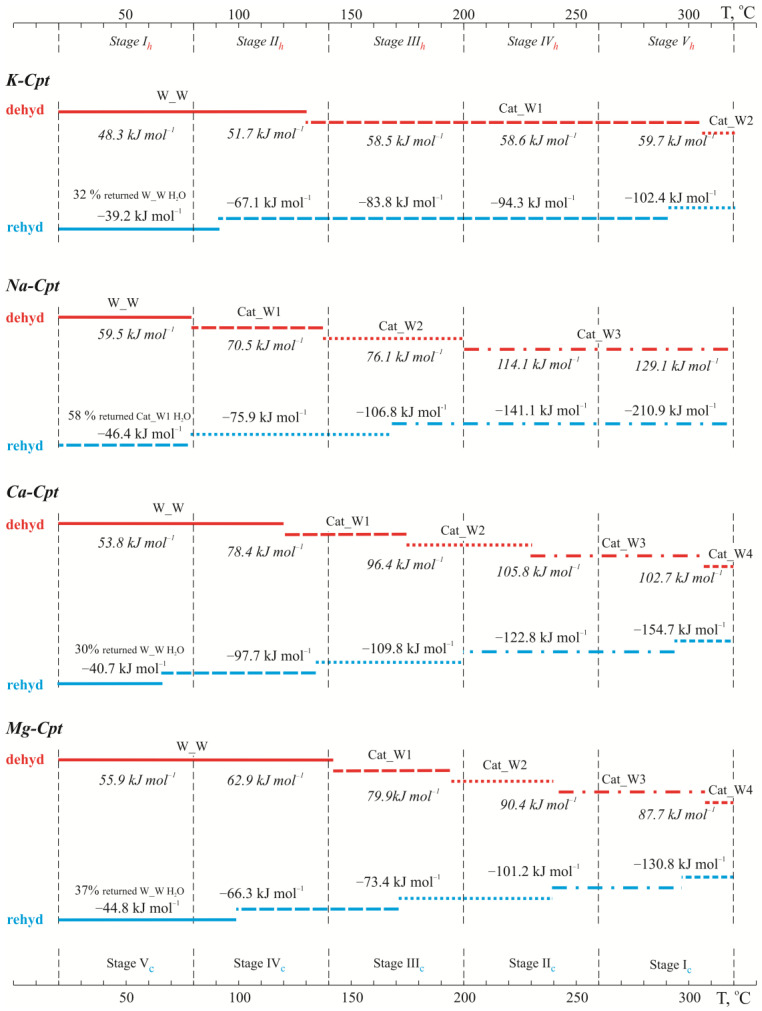
Comparison of rehydration-dehydration parameters in five stage experiment.

Under the experimental conditions, the rehydration process begins immediately with the start of cooling. The gradual return of individual water molecules extended over a larger temperature range (Figure 8), reaching a maximum degree of rehydration of 60–65% of the dehydrated water molecules (Table 5). In all samples, the rehydration completes at room conditions within 24 h.

The observed asymmetry of the reversible dehydration–rehydration process is due to the peculiarities of the experiment. On the one hand, equilibrium of the processes is not aimed and is not reached, and on the other hand, in the selected temperature ranges of the individual stages, the study is conducted in isothermal mode, at the high temperature of the range during heating and at the low temperature during cooling. However, clear trends are observed, caused by the influence of the extra-framework cation.

For example, although slightly pronounced, rehydration is more difficult in monovalent forms compared to divalent ones (Table 5), which is perhaps due to the more difficult movement of water molecules due to the two-fold greater number of cations located in the free space.

In terms of energy, in the first stages of cooling, the molar enthalpy values are unexpectedly high compared to the values obtained during dehydration (Figure 8). This can be explained by the high self-energy of the water molecules in the sorption medium (heated air), as with decreasing temperature, the values also decrease. On the other hand, a contribution to the high molar enthalpy during the sorption of the first molecules can also be expected from a decrease in the tension of the dehydrated structures. The values are especially high for the samples with a high degree of dehydration (K-Cpt and Na-Cpt). From the data obtained for the monovalent cationic forms, it is seen that the difference in the values of the molar enthalpy of desorption and sorption remains very high, mainly for the last water molecule coordinating the cation and its return as the first molecule. It is known that during complete dehydration, the extra-framework cations move to new positions, located closely to the framework [20]. During the interaction of the first adsorbed water molecules with the cations, these stronger bonds of the cations with the framework are apparently destroyed, which releases additional energy. This statement is supported by the recorded increase in the temperature of cooled thermally activated zeolites in the initial stages of wetting with water at room conditions, e.g., by 45–50 °C for natural clinoptilolite and 75–80 °C for synthetic Na-X [49].

In samples with a predominance of divalent extra-framework cations (Ca-Cpt and Mg-Cpt), the more inhomogeneous cation composition makes it difficult to assess where the first water molecules are adsorbed. In both samples, there is a significant amount of K cations (and in Ca-Cpt, and a noticeable amount of Na^+^), which, in the studied temperature range, are completely dehydrated. Therefore, the question arises whether the first entering molecules join the existing, coordinated with the two predominant divalent cations, water molecules (as formally shown in Figure 8), or hydrate the fully dehydrated monovalent cations.

Due to the specificity of the experiment, the obtained quantitative and thermo-technical data on rehydration are inapplicable to conventional drying at room conditions. Despite some value differences regarding the hydration enthalpy data obtained by other methodologies, they represent the first data on the sorption energy of sequentially attached water molecules in the dynamic cooling regime in the temperature range 320–20 °C.

## 3. Materials and Methods

### 3.1. Materials

Clinoptilolite tuff from the Beli Plast deposit, Eastern Rhodopes, Bulgaria, containing about 75% clinoptilolite, was used in this study. A purified form was obtained by removal of the cristobalite and the clay minerals and separation of the heavy minerals by centrifuge in a mixture of bromoform and acetone. The purified sample containing almost 98% clinoptilolite was exchanged with 1 M Na-, K-, Ca-, or Mg-chloride solution by shaking for three days at 60 °C, changing the solution each day, and finally washed and dried at 40 °C. The purification procedure of clinoptilolite tuff, the cation exchange procedure, and the chemical composition determination have been described before [35]. The clinoptilolite forms under this study have the following chemical composition:

As purified: (Na_0.61_K_1.90_Ca_1.32_Mg_0.35_)[(Al_5.83_Fe_0.06_ Si_30.12_)O_72_]∙24.1H_2_O
K-Cpt: (Na_0.64_K_5.39_Ca_0.04_Mg_0.13_)[(Al_5.76_Fe_0.05_Si_30.05_)O_72_]∙17.3H_2_O
Na-Cpt: (Na_4.65_K_1.05_Ca_0.06_Mg_0.18_)[(Al_5.53_Fe_0.06_Si_30.27_)O_72_]∙21.8H_2_O
Ca-Cpt: (Na_0.35_K_0.94_Ca_2.24_Mg_0.20_)[(Al_5.95_Fe_0.05_Si_29.96_)O_72_]∙23.9H_2_O
Mg-Cpt: (Na_0.05_K_1.4_ Ca_0.48_Mg_1.52_)[(Al_5.97_Fe_0.07_Si_30.12_)O_72_]∙23.8H_2_O

The above chemical compositions show a degree of cation exchange as follows: 84.8% in K-Cpt, 75.2% in Na-Cpt, 69.8% in Ca-Cpt, and 55.9% in Mg-Cpt.

### 3.2. Methods

TG and DSC analyses were performed on a Setsys Evolution 2400 analyzer, SETARAM, France, in a static air atmosphere. About 12 mg of the sample was placed in a corundum crucible, and another empty crucible was used as a reference. For the purpose of our study, three experimental approaches were used: (i) heating from 20 to 850 °C with a scanning rate of 10 °C min^−1^; (ii) multi stage approach containing four stages of heating (from 20 to 340 °C) and four stages of cooling (from 340 to 20 °C) with a scanning rate of 10 °C min^−1^ and an isothermal mode for 20 min through 80 °C during heating/cooling modes; (iii) five stages of heating (from 20 to 320 °C) and five stages of cooling (from 320 to 20 °C) with a scanning rate of 10 °C min^−1^ and an isothermal mode for 15 min through 60 °C during heating/cooling mode. An empty crucible run for baseline slope correction of both experimental TG and DSC signals was applied at each of the given set profiles. The first obtained derivative of the TG-curve (DTG) responds to changes in the rate of sample mass decreasing or increasing. Specialized CALISTO (1.6 version) thermal analysis software was used for conducting experiments and processing data.

## 4. Conclusions

Dehydration–rehydration processes of K-, Na-, Ca-, and Mg-exchanged forms of clinoptilolite were studied by applying a multi-stage TG-DSC approach with alternation of heating/holding and cooling/holding stages in the temperature range 20–320 °C. The influence of extra-framework cations on the parameters of dehydration and rehydration (temperatures, amounts of water molecules, molar enthalpy values, etc.) was established. (i) The dehydration of the different clinoptilolite forms shows that for the monovalent cationic forms, the dehydration is almost complete at a temperature of 320 °C, while for the bivalent forms, it is below 85%. In the dehydration process, water molecules sequentially evolved, characterized by different dehydration molar enthalpies. The data on the molar enthalpy is in good agreement with the distribution of W_W-type and Cat_W-type water in the applied crystal chemical gallery model of the cationic exchange samples used. This confirms the expediency of deriving W_W and Cat_W ensembles in HEU-type structures. Because the specific values of the molar enthalpy and their change amplitude in the individual stages are strongly influenced by the type of cations, the obtained mass and enthalpy data provide broad opportunities for comparing the potential for practical use of clinoptilolite forms from different deposits. The obtained data for water molecules and their molar enthalpies of dehydration for the various cationic forms are useful in studying the sorption of water vapor and other sorbates, in choosing a desiccant and an object to dry at room conditions, etc. (ii) The results of the rehydration show that the maximum degree of this reversible process under the experimental conditions is 60–65% of the dehydrated water molecules, and this process is more problematic for the monovalent forms compared to the divalent ones. In terms of energy, the values of the rehydration molar enthalpy in the first stages of cooling are much higher compared to the values obtained during dehydration. This phenomenon was explained by some crystal chemical considerations related to changes in the structure during dehydration, as well as by the high self-energy of the water molecules in the sorption medium. The first data on the sorption energy of sequentially added water molecules in a dynamic cooling mode in the temperature range 320–20 °C are obtained in this study.

## Figures and Tables

**Table 1:** Mass changes during the four-stage TG study for Cpt cationic exchanged forms.

Stage	Temperature Region, °C	K-Cpt		Na-Cpt		Ca-Cpt		Mg-Cpt	
		Mass Change (Δm, %)		Mass Change (Δm, %)		Mass Change (Δm, %)		Mass Change (Δm, %)	
		Heating	Cooling	Heating	Cooling	Heating	Cooling	Heating	Cooling
I	20–100	−5.68	+1.63	−5.38	+2.11	−5.57	+1.83	−5.55	+1.92
II	100–180	−3.21	+1.72	−4.24	+2.24	−3.09	+2.09	−3.96	+2.34
III	180–260	−1.88	+1.30	−2.27	+1.58	−2.08	+1.64	−2.31	+1.69
IV	260–340	−0.84	+0.40	−1.29	+0.77	−1.68	+1.09	−1.32	+0.98
Total	20–340	−11.61	+5.05	−13.18	+6.70	−12.42	+6.65	−13.14	+6.99

**Table 2:** Mass losses and heats of dehydration of cationically exchanged Cpt forms for dynamic and isothermal five heating stages.

Stage (T, °C)		I (20–80)		II (80–140)		III (140–200)		IV (200–260)		V (260–320)		Total (20–320)	
Sample	Mode	Δm (%)	Oz (J g^−1^)	Δm (%)	Oz (J g^−1^)	Δm (%)	Oz (J g^−1^)	Δm (%)	Oz (J g^−1^)	Δm (%)	Oz (J g^−1^)	Δm (%)	Oz (J g^−1^)
K-Cpt	dynamic	3.28	97.6	2.87	88.7	1.80	61.7	1.33	45.6	0.83	27.5	10.11	321.1
	isotherm	0.52	4.3	0.31	2.6	0.18	3.3	0.16	2.9	0.10	3.3	1.28	16.4
	Total	3.80	101.9	3.18	91.3	1.98	65.0	1.49	48.5	0.93	30.8	11.39	337.5
Na-Cpt	dynamic	2.99	113.3	3.49	148.3	2.56	112.6	1.44	90.2	1.07	71.7	11.55	539.7
	isotherm	0.50	1.7	0.44	5.7	0.29	2.5	0.11	6.8	0.12	6.5	1.46	19.5
	Total	3.49	115.0	3.93	154.0	2.85	115.1	1.55	97.0	1.19	78.2	13.01	559.2
Ca-Cpt	dynamic	3.21	100.5	3.36	153.6	1.91	106.1	1.42	85.1	1.22	70.7	11.13	516.4
	isotherm	0.50	3.3	0.30	5.5	0.19	4.8	0.16	7.8	0.22	6.8	1.36	27.7
	Total	3.71	103.8	3.65	158.9	2.10	110.9	1.58	92.9	1.44	77.5	12.49	544.1
Mg-Cpt	dynamic	2.96	103.2	3.10	116.6	2.46	100.1	1.41	75.5	1.0	49.8	10.94	445.2
	isotherm	0.45	2.6	0.34	3.6	0.18	2.4	0.21	5.2	0.09	3.3	1.27	17.0
	Total	3.41	105.8	3.44	120.2	2.64	102.5	1.62	80.7	1.09	53.1	12.00	462.2

**Table 3:** Theoretical distribution of water molecules in the specific samples studied according to the gallery model.

Sample	Number of H_2_O Molecules							
	W_W	Cat_W1	Cat_W2	Cat_W3	Cat_W4	Cat_W5	Cat_W6	Total H_2_O
K-Cpt	9.01	6.20	0.81	0.81	0.17	0.17	0.13	17.3
Coordinated at:								
K^+^ Na^+^ Ca^2+^ Mg^2+^		5.39 0.64 0.04 0.13	0.64 0.04 0.13	0.64 0.04 0.13	0.04 0.13	0.04 0.13	0.13	
Na-Cpt	5.42	5.94	4.89	4.89	0.24	0.24	0.18	21.8
Coordinated at:								
K^+^ Na^+^ Ca^2+^ Mg^2+^		1.05 4.65 0.06 0.18	4.65 0.06 0.18	4.65 0.06 0.18	0.06 0.18	0.06 0.18	0.18	
Ca-Cpt	9.51	3.73	2.79	2.79	2.44	2.44	0.20	23.9
Coordinated at:								
K^+^ Na^+^ Ca^2+^ Mg^2+^		0.94 0.35 2.24 0.20	0.35 2.24 0.20	0.35 2.24 0.20	2.24 0.20	2.24 0.20	0.20	
Mg-Cpt	10.73	3.45	2.05	2.05	2.0	2.00	1.52	23.8
Coordinated at:								
K^+^ Na^+^ Ca^2+^ Mg^2+^		1.40 0.05 0.48 1.52	0.05 0.48 1.52	0.05 0.48 1.52	0.48 1.52	0.48 1.52	1.52	

**Table 4:** Number of water molecules and molar enthalpy values obtained during their release in each experimental stage, consistent with the theoretical gallery-type structural model.

Sample H_2_O Type Theor. Model	nH_2_O in Theor. Model	Experimental Data for Stages											H_2_O
		I 25–80 °C		II 80–140 °C		III 140–200 °C		IV 200–260 °C		V 260–320 °C		Total 25–320 °C	Remained in the Structure
		nH_2_O	ΔH, kJ mol^−1^	nH_2_O	ΔH, kJ mol^−1^	nH_2_O	ΔH, kJ mol^−1^	nH_2_O	ΔH, kJ mol^−1^	nH_2_O	ΔH, kJ mol^−1^	nH_2_O	
K-Cpt		5.17	48.3	4.33	51.7	2.69	58.5	2.03	58.6	1.26	59.7	15.48	1.82
W_W	9.01	5.17		3.84									
Cat_W_1_	6.20			0.49		2.69		2.03		0.99			
Cat_W_2_	0.81									0.27			0.54
Cat_W3–6	1.28												1.28
Na-Cpt		5.52	59.5	6.22	70.5	4.51	76.1	2.46	114.1	1.77	129.1	20.48	1.32
W_W	5.42	5.42											
Cat_W1	5.94	0.10		5.84									
Cat_W2	4.89			0.38		4.51							
Cat_W3	4.89							2.46		1.77			0.67
Cat_W4–6	0.65												0.65
Ca-Cpt		5.75	53.8	5.67	78.4	3.26	96.4	2.45	105.8	2.23	102.7	19.36	4.54
W_W	9.51	5.75		3.76									
Cat_W1	3.73			1.91		1.82							
Cat_W2	2.79					1.44							
Cat_W3	2.79							1.10		1.69			
Cat_W4–6	5.08									0.54			4.54
Mg-Cpt		5.26	55.9	5.31	62.9	4.07	79.9	2.50	90.4	1.68	87.7	18.82	4.98
W_W	10.73	5.26		5.30		0.17							
Cat_W1	3.45					3.45							
Cat_W2	2.05					0.46		1.59					
Cat_W3	2.05							0.91		1.14			
Cat_W4–6	5.52									0.54			4.98

**Table 5:** Leaved and returned H_2_O molecules calculated based on the mass changes during dehydration and rehydration of different cationic samples.

		Dehydration		Rehydration	
Sample	Initial nH_2_O	Leaved nH_2_O	Degree of Dehydration, %	Returned nH_2_O	% of Rehydration (Compared to Dehydrated Samples)
K-Cpt	17.3	15.48	90	9.35	60
Na-Cpt	21.8	20.48	94	12.55	61
Ca-Cpt	23.9	19.36	81	12.73	66
Mg-Cpt	23.8	18.82	79	12.01	64

## Data Availability

Data is contained within the article.

## Abbreviations

The following abbreviations are used in this manuscript:

Cpt	clinoptilolite
HEU	Heulandite type structure
W_W	Water-water assemblages
Cat_W	cation–water assemblages
TG	Thermogravimetry
DTG	Differential Thermogravimetry
DSC	Differential Scanning Calorimetry
